# Cardiorespiratory fitness as a predictor of intestinal microbial diversity and distinct metagenomic functions

**DOI:** 10.1186/s40168-016-0189-7

**Published:** 2016-08-08

**Authors:** Mehrbod Estaki, Jason Pither, Peter Baumeister, Jonathan P. Little, Sandeep K. Gill, Sanjoy Ghosh, Zahra Ahmadi-Vand, Katelyn R. Marsden, Deanna L. Gibson

**Affiliations:** 1 Department of Biology, The Irving K. Barber School of Arts and Sciences, University of British Columbia, Room ASC 386, 3187 University Way, Okanagan campus, Kelowna, British Columbia V1V 1V7 Canada; 2 School of Health and Exercise Sciences, University of British Columbia, Okanagan campus, Kelowna, British Columbia V1V 1V7 Canada

**Keywords:** Intestinal microbiota, Microbial ecology, Physical activity, Exercise, Butyrate, Community diversity, Metagenome, Dysbiosis

## Abstract

**Background:**

Reduced microbial diversity in human intestines has been implicated in various conditions such as diabetes, colorectal cancer, and inflammatory bowel disease. The role of physical fitness in the context of human intestinal microbiota is currently not known. We used high-throughput sequencing to analyze fecal microbiota of 39 healthy participants with similar age, BMI, and diets but with varying cardiorespiratory fitness levels. Fecal short-chain fatty acids were analyzed using gas chromatography.

**Results:**

We showed that peak oxygen uptake (VO_2_peak), the gold standard measure of cardiorespiratory fitness, can account for more than 20 % of the variation in taxonomic richness, after accounting for all other factors, including diet. While VO_2_peak did not explain variation in beta diversity, it did play a significant role in explaining variation in the microbiomes’ predicted metagenomic functions, aligning positively with genes related to bacterial chemotaxis, motility, and fatty acid biosynthesis. These predicted functions were supported by measured increases in production of fecal butyrate, a short-chain fatty acid associated with improved gut health, amongst physically fit participants. We also identified increased abundances of key butyrate-producing taxa (*Clostridiales*, *Roseburia*, *Lachnospiraceae*, and *Erysipelotrichaceae*) amongst these individuals, which likely contributed to the observed increases in butyrate levels.

**Conclusions:**

Results from this study show that cardiorespiratory fitness is correlated with increased microbial diversity in healthy humans and that the associated changes are anchored around a set of functional cores rather than specific taxa. The microbial profiles of fit individuals favor the production of butyrate. As increased microbiota diversity and butyrate production is associated with overall host health, our findings warrant the use of exercise prescription as an adjuvant therapy in combating dysbiosis-associated diseases.

**Electronic supplementary material:**

The online version of this article (doi:10.1186/s40168-016-0189-7) contains supplementary material, which is available to authorized users.

## Background

The interactions between humans, their environment, and intestinal microbiota form a tripartite relationship that is fundamental to the physiological homeostasis and overall health of the host [1]. The human intestinal microbiota aids their host in several important biological functions such as digestion, absorption, stimulating immune responses, and protection against enteropathogens. The bacteria break down partially digested complex carbohydrates via fermentation and produce short-chain fatty acids (SCFAs) such as butyrate, acetate, and propionate as by-products. These SCFAs act as the primary food source of the colonocytes which consume up to 10 % of the dietary energy expenditure in humans. In particular, butyrate has been shown to play a critical role in overall gut homeostasis and health [2]. Lasting disturbances in the microbial community composition, termed dysbiosis, can have deleterious health effects in the host (reviewed in [3]). Gut microbiome diversity has emerged as a candidate indicator of overall host health. Low community richness has been correlated with metabolic markers such as adiposity, insulin resistance, and overall inflammatory phenotypes [4], as well as gastrointestinal (GI) conditions such as inflammatory bowel disease [5], *Clostridium difficile* infection [6], colorectal cancer [7], and irritable bowel syndrome [8]. As a result, considerable research in recent years has focused on understanding and developing strategies to promote overall GI health via community manipulation in attempt to resolve dysbiosis-associated diseases.

Various extrinsic variables such as stress, probiotic and antibiotic use, alcohol consumption, and diet have been identified as factors that can instigate changes in the microbiome [1, 9]. The link between physical activity and gut microbiota however is currently not well understood. Matsumoto et al. (2008) first identified increases in butyrate levels in cecum of physically active rats which they suggested was a result of compositional changes in butyrate-producing bacteria [10]. Evans et al. explored the effects of voluntary wheel running in mice fed with low- or high-fat diets and found that microbial communities clustered based on both diet and physical activity [11]. Allen et al. further showed that the mode of physical activity, for example, forced treadmill running versus volunteer wheel running, differently altered the microbiota [12]. Recently, Clarke et al. also found clustering of bacterial communities between professional rugby players and high/low body mass index (BMI) controls [13]. They further identified increases in bacterial community richness in these elite athletes compared to both control groups. In their study, however, extreme dietary differences, especially high protein intakes amongst the athletes, confounded interpretations regarding the specific role of physical activity and microbial changes.

To better isolate how physical fitness may moderate microbial diversity, we analyzed the fecal microbiota of individuals with varied fitness levels with comparable diets. We used peak oxygen uptake (VO_2_peak), the gold standard of cardiorespiratory fitness (CRF), as an indicator of physical fitness. We asked the questions (a) does taxonomical richness vary with CRF alone, (b) do abundances of particular taxa vary systematically in relation to variation in CRF, and (c) is this variation associated with functional pathways of the microbiome. We show that VO_2_peak, independent of diet, correlates with increased microbial diversity and production of fecal butyrate amongst physically fit participants.

## Methods

### Study design

Healthy young adults between 18 and 35 years old were recruited. Exclusion criteria included antibiotic treatment within the previous 6 months, current prescribed pharmaceutical drug utilization, or active acute or chronic diseases. All participants were verbally interviewed on their dietary habits and CRF was determined using a VO_2_peak cycle test. Participants were then provided a stool collection kit with instructions and were asked to provide a sample within a week following their lab visit.

### Nutritional data collection

On the day of VO2peak testing, nutritional data, including supplements, was collected by means of a 24-h dietary recall interview and assessed by a research nutritionist using FoodWorks nutrient analysis software (version 16.0). Food items described by participants that were not available in the software were manually added as needed. A sample copy of a completed questionnaire is available in Additional file 1. On average, over 100 food categories per participant were produced by the FoodWorks software. A manual screening was applied to select categories of interest based on a priori interest and existing literature showing a significant interaction between those categories and intestinal microbiota. The selected 24 food category data are available in the uploaded metadata mapping file.

### Cardiorespiratory fitness testing

Participants initially completed a physical activity readiness questionnaire (PAR-Q) to rule out any contraindications to vigorous exercise. A continuous incremental ramp maximal exercise test on an electronically braked cycle ergometer (Lode Excalibur, the Netherlands) was used to determine VO_2_peak and peak power output (Wpeak). Expired gas was collected continuously by a metabolic cart (Parvomedics TrueOne 2400, Salt Lake City, Utah, USA) calibrated with gases of known concentration. The test started at 50 W and increased by 30 W/min. The test was terminated upon volitional exhaustion or when revolutions per minute fell below 50. VO_2_peak was defined as the highest 30-s average for VO_2_ (in ml/kg/min). Criteria for achieving VO_2_peak were the following: (i) respiratory exchange ratio >1.15; (ii) plateau in VO_2_; (iii) reaching age-predicted HRpeak (220-age); and/or (iv) volitional exhaustion. Following VO_2_peak assessment, participants were categorized to either low (LOW), average (AVG), or high (HI) fitness based on their sex and age according to a modified Heyward normal VO_2_max reference chart (Additional file 2).

### Stool collection and storage

Participants were provided with a home stool collection kit including a sterile 120 ml polypropylene container (Starplex, Etobicoke, Ontario), sterile tongue depressor and gloves, and an ice box. Participants were instructed to avoid alcohol for 3 days prior to stool collection. Stool samples were immediately stored in the participant’s freezer overnight and transported on ice to the lab and stored in −80 °C until further analysis. Frozen portions from the inner area of the samples were scrapped using sterile razor blades for DNA extraction and SCFA analysis.

### SCFA analysis

SCFAs (acetic, propionic, heptanoic, valeric, caproic, and butyric acid) were analyzed from the feces by gas chromatography (GC) as described previously [14]. In brief, ~50 mg of stool was homogenized with isopropyl alcohol, containing 2-ethylbutyric acid at 0.01 % *v*/*v* as internal standard, at 30 Hz for 13 min using metal beads. Homogenates were centrifuged twice, and the cleared supernatant was injected to Trace 1300 Gas Chromatograph, equipped with Flame-ionization detector, with AI1310 auto sampler (Thermo Fisher Scientific) in splitless mode. Data was processed using Chromeleon 7 software. An aliquot of 50 mg of stool was freeze dried to measure the dry weight, and measurements are expressed as mass % (g of SCFA per g of dry weight stool).

### High-throughput sequencing

DNA was extracted from feces using QIAmp DNA Stool Mini Kit (Qiagen) according to the manufacturer’s instructions following 3 × 30 s of homogenization using metal beads on a Retsch MixerMill MM 400 homogenizer. Sequencing libraries were prepared according to the Illumina MiSeq system instructions. In brief, the V3 and V4 region of the 16S bacterial rRNA gene was amplified using recommended primers [15] (IDT, Vancouver, Canada): Forward 5′ TCGTCGGCAGCGTCAGATGTGTATAAGAGACAGCCTACGGGNGGCWGCAG, and Reverse 5′ GTCTCGTGGGCTCGGAGATGTGTATAAGAGACAGGACTACHVGGGTATCTAATCC, which create amplicons of ~460 bp. Amplicons were cleaned using AMPure XP bead step, and then, adapters and dual-index barcodes (Nextera XT) were attached to the amplicons to facilitate multiplex sequencing. After another clean-up step, libraries were validated on an agarose gel, quantified, normalized, and sent to The Applied Genomic Core (TAGC) facility at the University of Alberta (Edmonton, Canada) for sequencing using the Illumina MiSeq platform. The resulting ~16,000,000 paired-end reads were merged using PEAR software [16] and screened to exclude sequences containing one or more base calls with a Phred score <20. The average read per sample was ~350,000 with a min/max of ~165,000/452,000 reads. Rarefaction curves demonstrated that sufficient sampling depth had been reached amongst all samples (Additional file 3).

### Bioinformatics

Bioinformatics analyses on the demultiplexed paired reads were conducted using QIIME 1.8.0 software suites [17]. Reads were clustered at 97 % identity using the *uclust* method into operational taxonomic units (OTUs) then aligned to the most recent available version (2013/08) of Greengenes bacterial database [18]. Singleton and doubletons were removed, and the produced OTU table was normalized using phylogenetic investigation of communities by reconstruction of unobserved states (PICRUSt) [19] to adjust for different 16S rRNA gene copy numbers. Instead of rarefying our OTU table to the lowest sample depth [20], uneven variance as a result of differential sample sequencing depth was stabilized using the cumulative sum scaling (CSS) method [21] of “metagenomeSeq” package in R. Alpha diversity indexes, rarefaction curves, OTU tables, and distance metrics were also generated using QIIME.

### Statistical analysis

All statistical analyses were performed using R [22] version 3.2.0 unless stated otherwise.

The groups’ age and VO_2_peak data were tested for normality using Shapiro-Wilk test, and a one-way analysis of variance (ANOVA) with Tukey’s multiple-comparison test was used to compare mean differences amongst groups. Kruskal-Wallis non-parametric test was used for comparing BMI as this dataset failed normality tests even after several transformation attempts. For comparison of dietary intake amongst groups, a permutational multivariate ANOVA (PERMANOVA) with 999 random permutations was used. Due to the inherent high variability of dietary data, we further searched for dietary patterns amongst groups by looking at a principal component analysis (PCA) plot of participants’ dietary scores using the *ggbiplot* package [23]. To facilitate comparisons with previous work, we first compared average alpha diversity amongst the three fitness categories using a one-way ANOVA, followed by a Tukey’s multiple comparison. To simultaneously evaluate the role of CRF alongside other potential predictors of alpha diversity (sex, age, BMI, and dietary components), we performed a multiple regression analysis. Given our comparatively low sample size (*n* = 39), and the general rule that multiple regressions should include at least 10 observations per predictor variable [24], we first screened potential predictors using a Spearman correlation matrix. Those that showed a significant correlation with alpha diversity were retained for entry in the multiple regression model. Multicolinearity was checked using the variable inflation factor (VIF) index with a maximum cutoff score of 10.

Microbial communities in fecal samples were ordinated using the Bray-Curtis and weighted and unweighted UniFrac distance metrics. Principal coordinate analysis (PCoA) based on the Bray-Curtis dissimilarity metric was conducted using the *cmdscale* function in the base “stats” package in R, while PCoA based on the weighted and unweighted unifrac distances was made using EMPeror tool [25]. Microbial communities were analyzed using two complementary multivariate approaches: (1) constrained ordination and (2) generalized linear models (GLM). For the constrained ordination approach, redundancy analysis (RDA) was used, which focuses on assemblage composition differences in relation to predictors of interest (VO_2_peak, sex, age, BMI, and dietary components). This was implemented using the “vegan” package [26] version 2.2-1 in R. Abundance data at each taxonomical resolution (phyla, class, order, family, and genus) were first Hellinger-transformed [27] to accommodate counts data with large occurrences of low and zero abundance. Variable selection in RDA was implemented using the *ordistep* function of vegan using both forward and backward stepwise inclusion. Predictors selected by this method at each classification level are presented in Additional file 4. To identify genera that significantly contributed to total variance, we evaluated Spearman correlations between genus abundance and the first two RDA axes. OTUs with a significant correlation coefficient (evaluated at Bonferroni adjusted alpha level) were displayed on the RDA plots with type II scaling. To evaluate the association of genus abundance with explanatory variables, we implemented multiple negative binomial GLMs using the “mvabund” package [28]. This multiple GLM method utilizes a series of univariate *F* tests of the effects of predictor variables on the abundance of each taxon. Regression assumptions were assessed using residual diagnostics. Taxa that made up less than 0.1 % of the total count and occurring in less than 75 % of samples were first removed (cf. [29]). Fifty taxa met the inclusion criteria and were included in the model. The default implementation of the multi-GLM method adjusts *P* values to account for multiple tests. Classification of relative abundance data according to the previously described enterotypes [30] was carried out using the Calinski-Harabasz (CH) index as described online (http://enterotype.embl.de/enterotypes.html).

Bacterial phylogeny is sufficiently linked to their functional capabilities and can be used to computationally predict the functional composition of the community metagenome [19]. The normalized genus abundance OTU table was used to predict the microbiome’s metagenomic functions using PICRUSt’s extended ancestral-state reconstruction approach. A new abundance matrix of predicted functional categories based on the Kyoto Encyclopedia of Genes and Genomes (KEGG) database was created. We constructed a biplot from the output of a PCA of functional category data and visually assessed clustering patterns based on CRF groupings. Next, to isolate the influence of specific predictor variables, an RDA was also performed using these functional categories as response variables and the same variables and selection methods described above.

Similarly, to determine the role of our exploratory variables in explaining variance in fecal SCFAs, an RDA was conducted using SCFA abundance data as the response variables (cf. [31]).

## Results

### Diet was not a confounding factor across fitness groups

**Fig. 1:**
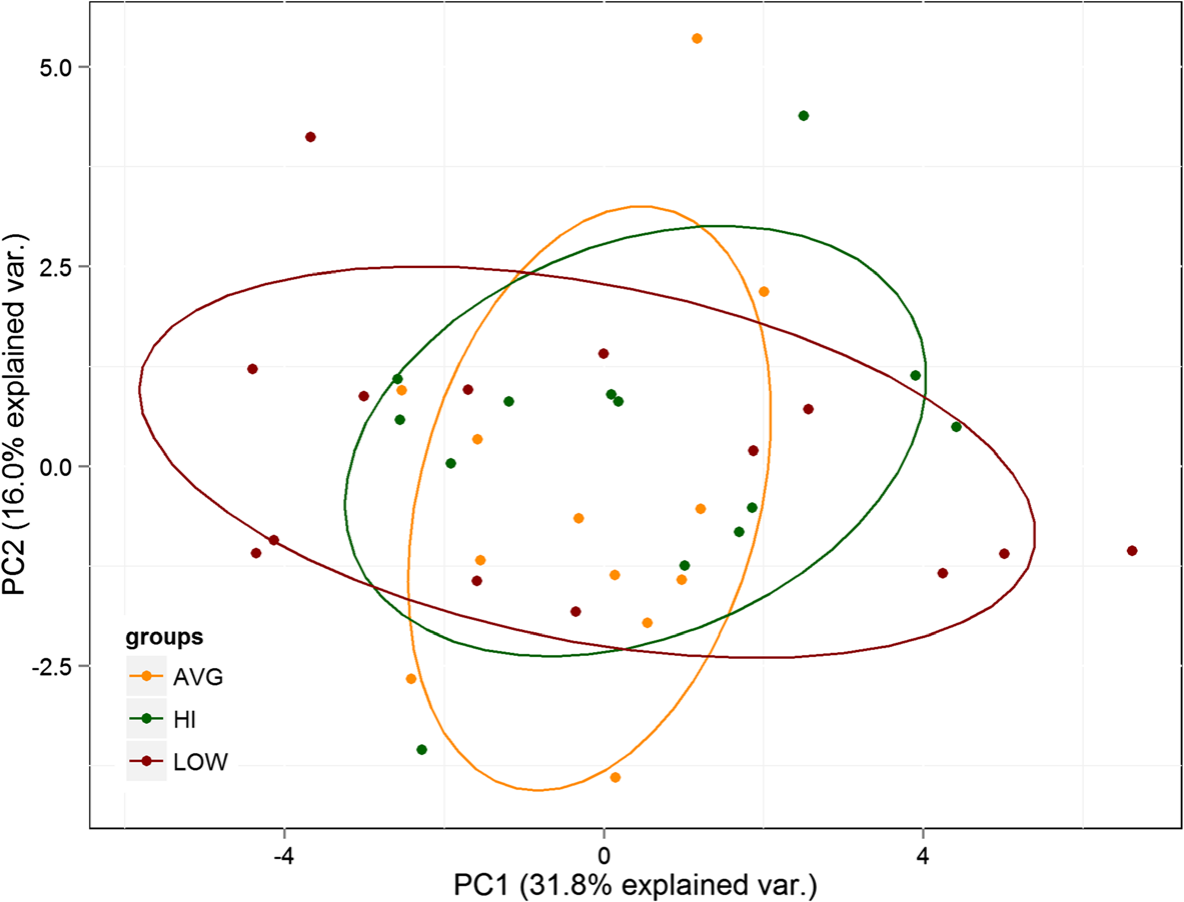
Dietary patterns amongst fitness groups. Scores of the two first components of the PCA of dietary data for all 39 subjects are presented. *Each circle* represents one participant, colored based on their CRF fitness levels. A lack of distinct clustering amongst groups suggests comparable dietary patterns amongst groups

Twenty-two males and 19 females participated in the study. Two female participants were removed from sequencing analysis due to technical errors. Table 1 represents a summary of the 39 participants’ characteristics and dietary intake. Only one participant followed a vegetarian diet, and all 39 participants reported consuming dairy products (data not shown). Age distribution was similar across all groups. The LOW group had a marginally higher BMI (25.5, SD 3.9) compared to the AVG (23.5, SD .5) and HI (22.8, SD 1.5) groups; however, the difference was not statistically significant. BMI of AVG and HI groups falls within the “normal weight” range (18.5–24.9) as defined by Health Canada, while the LOW group is marginally above the “overweight” threshold of 25. The results of the PERMANOVA (Additional file 5) showed no main differences (permutation *P* = 0.56) across any nutritional classes based on fitness groups. PCA plot (Fig. 1) of dietary patterns amongst the different fitness groups also showed no discrete clusters, further supporting a lack of distinct dietary patterns amongst fitness groups.

**Table 1:** Summary of group characteristics and dietary intake

	LOW (*n* = 14)		AVG (*n* = 12)		HI (*n* = 13)	
	Mean (SD)	Median (IQR)	Mean (SD)	Median (IQR)	Mean (SD)	Median (IQR)
Age (years)	25.5 (3.3)	25.5 (23–27.8)	24.3 (3.7)	24.5 (21.8–26)	26.2 (5.5)	28 (21–31)
BMI (kg/m^2^)	25.5 (3.9)	24.9 (23.2–27.8)	23.5 (.5)	23.4 (22.1–23.8)	22.8 (1.5)	22.4 (21.9–24)
VO_2_peak	33 (4.8)*	33.3 (30.7–26.3)	41.9 (4.3)*	41.2 (38.5–44.2)	54.8 (5.6)*	52.4 (51.3–60.9)
Dietary components						
Energy (kcal)	2477.5 (1168.4)	2119.5 (1537.2–3565)	2230 (605.4)	2092 (1793–2561)	2458.3 (668.3)	2647 (2060–2714)
Protein (g)	128.7 (88.5)	104.8 (55.4–182.7)	110.2 (53.7)	90 (80–134.6)	111.2 (49.7)	97.5 (84–127.2)
Carbohydrate (g)	278.9 (97.5)	294.7 (201.7–347.6)	245.2 (90.4)	245.2 (182.1–275.2)	276.9 (80.2)	268.5 (248.3–310.8)
Fat (g)	95.4 (61.9)	74.1 (46.5–121.3)	95.8 (29.1)	85.6 (78.4–113.6)	105.3 (41.1)	111.9 (84.2–131.30)
Saturated fat (g)	37.7 (30)	25.2 (16.9–62.2)	32 (29.1)	31.2 (26.5–34.5)	31.6 (14.7)	32.6 (21.2–36)
MUFA (g)	30.7 (19.9)	27.6 (14.1–36.9)	35 (14.4)	34.9 (27.6–40.8)	38.6 (16.5)	36.4 (28.5–46.9)
PUFA (g)	15 (6.8)	15.1 (9.3–20)	20.2 (11.7)	17.9 (11.1–26.7)	23.3 (10.7)	22.6 (15.4–28.2)
Trans fat (mg)	730 (960)	358 (28.5–89.3)	580 (440)	552 (243.7–896.8)	500 (530)	407 (87–501)
Omega 3 (mg)	2260 (1470)	1958 (−1166–3068)	2990 (2320)	1958 (1307–4779)	3110 (3600)	1535 (1200–1942)
Omega 6 (mg)	1790 (3320)	418 (28.8–1624)	1010 (1040)	438 (283.3–1672)	3820 (4250)	2477 (198–4951)
Sugar (g)	96.7 (59.1)	68.9 (54.8–134.2)	83.2 (43.9)	80.2 (67–95.5)	103.6 (38.4)	97.4 (81.7–121.7)
Fiber (g)	28.4 (11.7)	22.5 (20.2–34.7)	31.3 (30.2)	23.2 (17.3–29.4)	36.5 (20.2)	28.8 (24.2–40.2)
Cholesterol (mg)	358 (348.7)	263.6 (59.4–453.4)	346.5 (194.6)	288.6 (196.1–466.1)	443.1 (269.3)	442.6 (186.3–638.6)
Butyrate (mg)	470 (740)	212.5 (39.8–578)	690 (690)	573.5 (283.5–929)	480 (470)	366 (194–518)

As described fully under the methods section, dietary components amongst groups were compared by PERMANOVA, BMI comparison utilized Kruskal-Wallis test, while VO_2_peak and age were compared using a 1-way ANOVA test

*BMI* body mass index, *MUFA* monounsaturated fatty acid, *PUFA* polyunsaturated fatty acid, *SD* standard deviation, *IQR* interquartile range

*A significant (*P* < 0.01) pairwise difference amongst the other two groups using a Bonferroni alpha correction procedure

#### CRF is correlated with increased microbial diversity

**Fig. 2:**
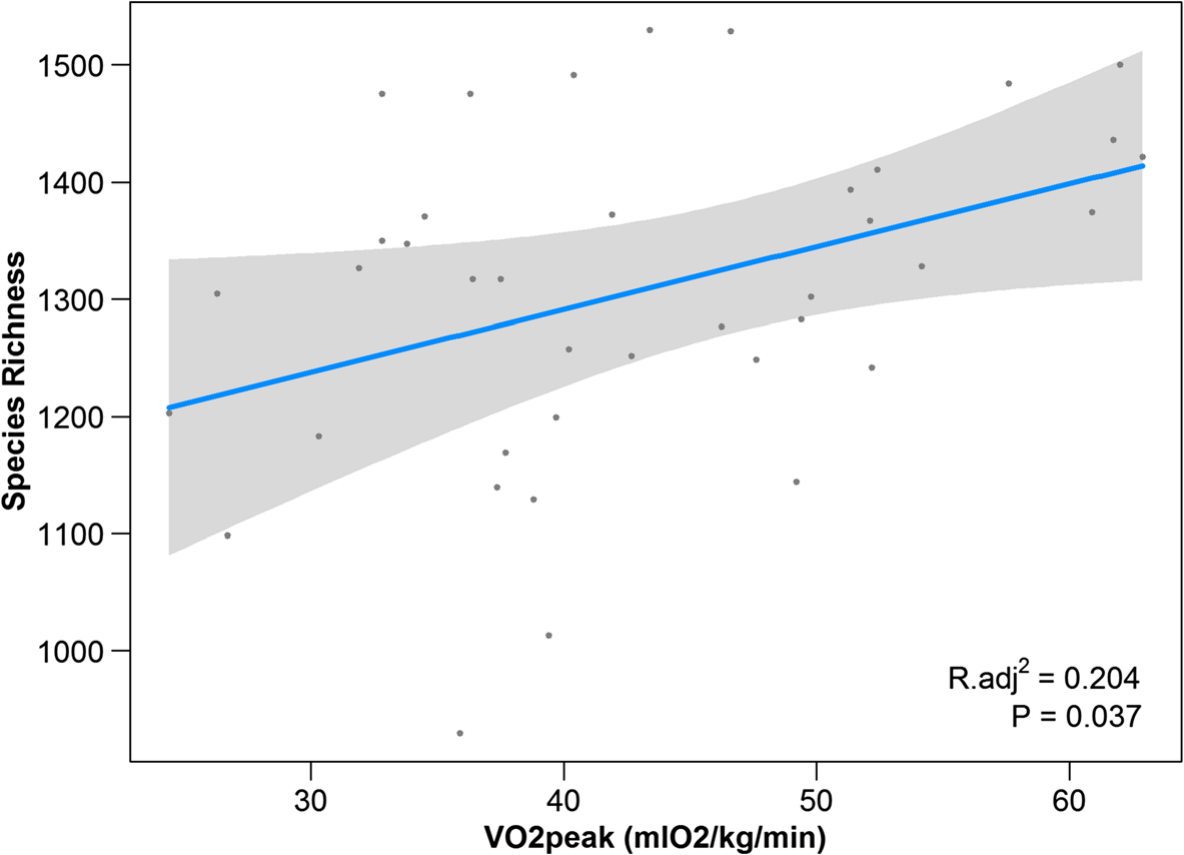
Correlation between VO_2_peak and species richness (SR). Result of a multiple regression model showing a significant association between VO_2_peak and SR when holding all other variables constant. *Shaded area* represent 95 % confidence intervals

Species diversity of each participant (alpha diversity) was determined using several indexes: species richness (SR), chao1, Shannon, Simpson, and Faith’s phylogenetic diversity (PD). As all the alpha diversity indexes were highly correlated (Additional file 6), SR was chosen as a proxy in the regression model. After screening of potential predictors via Spearman correlation analysis, three variables were included in the multiple regression model: VO_2_peak, sex, and relative fat intake. Of these, only VO_2_peak was a significant predictor of alpha diversity (Table 2), with SR significantly (*P* = 0.011) associated with increasing VO_2_peak (*R*_adj_^2^ = 0.204, coefficient estimate = 5.36, *t* = 2.17) (Fig. 2). Replacing SR with chao1, Shannon, and Simpson index in the regression model produced identical results in that VO_2_peak was the only significant predictor of these indices. Faith’s PD index showed a similar relationship with VO_2_peak (Pearson’s *R* = 0.30, *P* = 0.062); however, it did not reach statistical significance within the regression model (*R*_adj_^2^ = 0.07, coefficient estimate = 0.14, *t* = 1.12).

**Table 2:** Multiple regression summary table of species richness data

Variables	Unstandardized coefficients		Standardized coefficients	*t*	*P*
	B	Std. error	Beta		
VO_2_peak	5.36	2.47	0.37	2.17	0.037*
Relative fat intake	432.46	250.10	0.26	1.72	0.094
Sex^♂^	24.70	51.23	0.08	7.54	0.63

Result of multiple regression test showing VO_2_peak as the only significant variable in predicting species richness (SR). The B coefficient represents the amount of change in SR along its 95 % confidence intervals per unit change of VO_2_peak (ml/kg/min). The standardized coefficients show VO_2_peak as the strongest variable to influence SR variability. Model adjusted *R* ^2^ = 0.20. *P* value = 0.01

*Statistical significance

#### CRF levels do not promote distinct clustering of beta diversity data

**Fig. 3:**
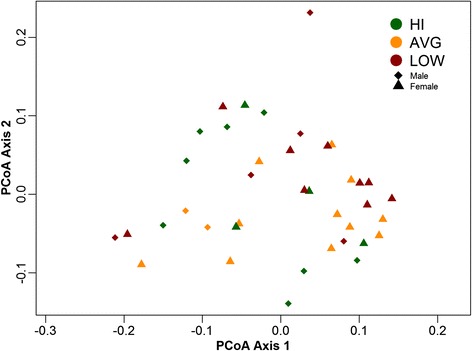
Beta diversity amongst fitness groups. PCoA plot of genus abundance data based on Bray-Curtis dissimilarity measure shows no clear clustering when grouped according to CRF levels

Overall, 207 genera from 14 phyla were represented across all participants (Table 3). The HI group included representation from 173 genera, while the AVG and LOW groups were made up of 152 and 153, respectively. PCoA plots constructed using Bray-Curtis (Fig. 3); weighted and unweighted unifrac dissimilarity indices (Additional file 7) did not show group clustering based on fitness levels. Clustering of our dataset based on the CH index favored a two cluster partitioning (Additional file 8) rather than the proposed three enterotypes [30].

**Table 3:** Identified known taxa across fitness groups

	LOW	AVG	HI	Total
Phylum	14	12	11	14
Class	28	23	23	31
Order	44	38	41	52
Family	76	73	79	92
Genus	153	152	173	207

Summary of the number of identified taxa across all participants as categorized based on their VO_2_peak levels

#### Protein intake and age but not CRF explain overall community composition

**Fig. 4:**
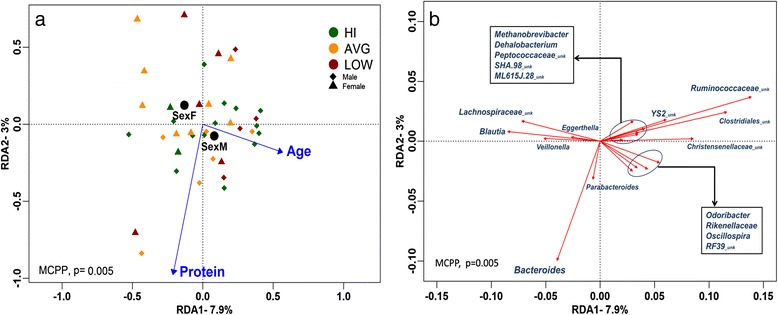
Bacterial abundance RDA correlation biplots constrained by selected explanatory variables. The sites and explanatory variables (**a**) and genera (**b**) plots are presented separately for clarity; however, they are derived from the same RDA model, note the difference in axes scales. RDA1 and RDA2 which explain over 10 % of total variation in beta diversity are plotted. The global model’s *P* value was calculated using the Monte Carlo Permutation Procedure (MCPP). In plot A, subjects are color coded according to their CRF levels for illustrative purposes only as groupings were not included in the model. *Black circles* represent centroids for the categorical variable sex

The global RDA model which selected sex, age, and protein as meaningful explanatory variables was significant (*P* = 0.005) as assessed by Monte Carlo Permutation Procedure (MCPP) (1000 permutations). A total of 12.7 % of the overall variation in taxon composition was attributed to these explanatory variables, of which the majority were explained by the first and second axes (Fig. 4) which accounted for 7.9 and 2.3 % of the total variation, respectively. The RDA indicated that VO_2_peak did not significantly explain beta diversity at any taxonomic resolution, whereas total protein intake was significant at each resolution tested (Additional file 4). In addition, age, sex, and the omega6-omega3 ratio (n6:n3) were also marginally significant explanatory variables, though only at particular taxanomic resolutions. In Fig. 4b, we highlight 19 genera that were significantly correlated with one or both of the first two RDA axes. Amongst these, *Bacteroides* was strongly associated with protein intake along RDA2 while *Odoribacter*, *Rikenellaceae*, *Oscillospira*, and an unclassified *RF39* were most strongly correlated with age along RDA1. Other genera that strongly aligned with RDA1, but not correlated with any explanatory variables, included *Blautia* and unclassified genera from *Lachnospiraceae*, *Christensenellaceae*, *Ruminococcaceae*, and *Clostridiales*.

#### CRF is associated with distinct microbiome functions rather than abundances of specific bacterial taxa

**Fig. 5:**
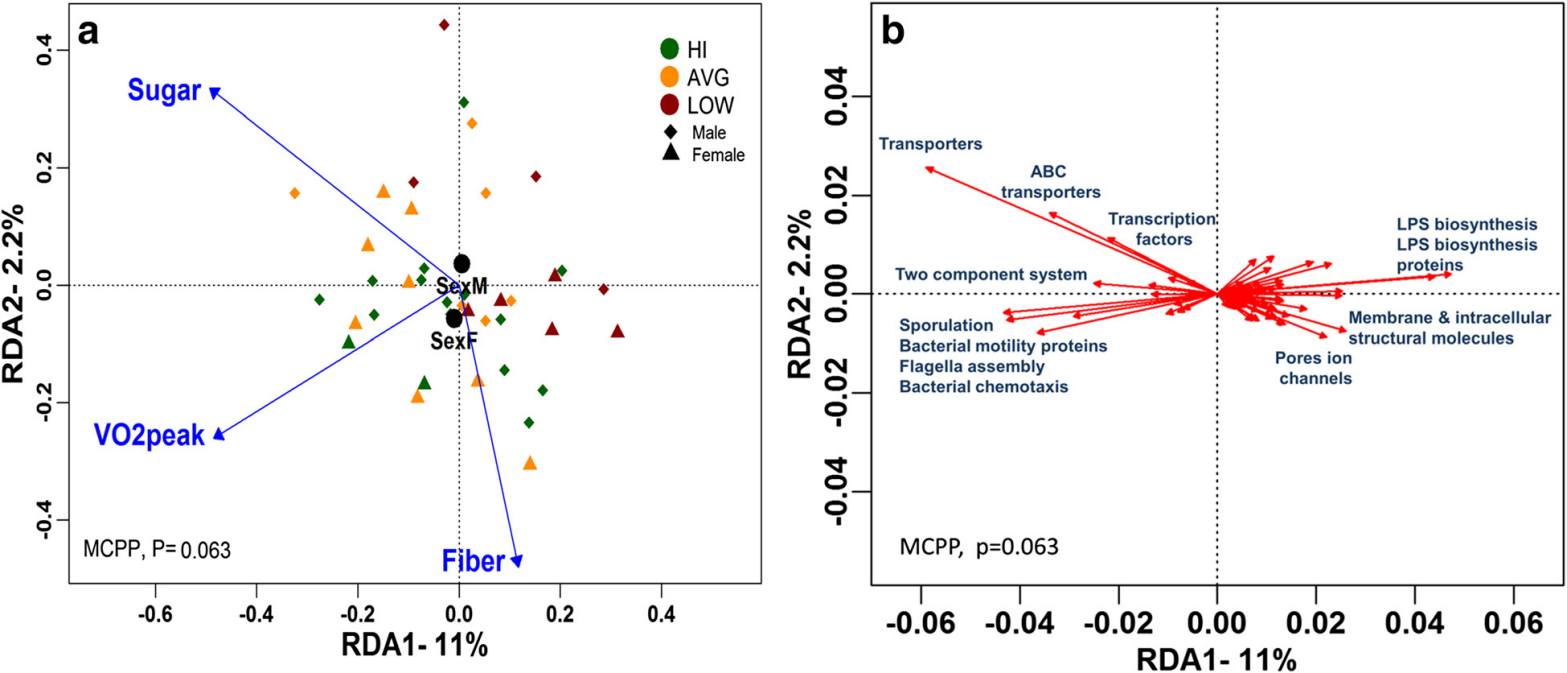
RDA correlation biplots of predicted metagenomics functions constrained by selected explanatory variables. The sites and explanatory variables (**a**) and genera (**b**) plots are presented separately for clarity; however, they are derived from the same RDA model, note the difference in axes scales. RDA1 and RDA2 which explain over 13 % of the total variation in data are plotted. The global model’s *P* value was calculated using the Monte Carlo Permutation Procedure (MCPP). In plot A, subjects are color coded according to their CRF for illustrative purposes only as groupings were not included in the model. *Black circles* represent centroids for the categorical variable sex

**Fig. 6:**
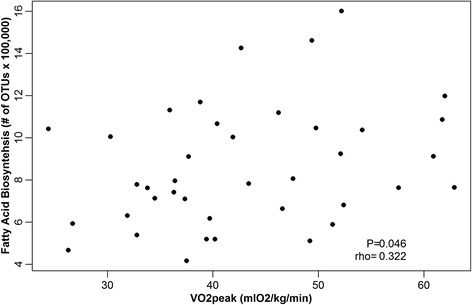
Correlation between VO_2_peak and fatty acid biosynthesis. Spearman correlation plot showing a positive relationship between VO_2_peak and the functional category “fatty acid biosynthesis.” *rho* Spearman’s correlation coefficient

Results of our GLMs suggest that, overall, genus abundances vary significantly in relation to our exploratory variables (model *P* = 0.003) with VO_2_peak and sex identified as significant factors (*P* < 0.002). After stringent adjustments for multiple testing, the univariate follow-up tests revealed no significant response amongst the 50 individual taxa included. Without adjusting for multiple testing (and keeping in mind the increased potential for type-1 errors), several taxa exhibited positive relationship with VO_2_peak (*P* < 0.05). These include, *Coprococcus*, *Roseburia*, *Adlercreutzi*a, and unknown members of *Clostridiales*, *Lachnospiraceae*, and *Erysipelotrichaceae*. We further explored whether the functional composition of the microbiomes were associated with CRF. Similar to the beta diversity analyses, no clear group clustering emerged based on CRF classification alone (Additional file 9). The RDA, however, showed that the variables VO_2_peak, sex, fiber, and sugar intake collectively had a marginally significant role in explaining compositional variation in functional categories (MCPP *P* = 0.063) (Additional file 4). Overall, 15.5 % of the total variation of the functional category composition was accounted for by these explanatory variables, of which 11 and 2.2 % were accounted for by the first and second axes, respectively (Fig. 5a, b). Of the 274 functional categories observed across all participants, we identified 65 significant categories. A complete list of the 65 identified functional categories is presented in Additional file 10. The RDA plots illustrate a pattern of VO_2_peak and fiber intake explaining variation amongst participants with high CRF levels. VO_2_peak was most strongly correlated with KEGG functional categories: sporulation, bacterial motility proteins including proteins involved in flagella assembly, and chemotaxis while negatively correlated with lipopolysaccharide (LPS) biosynthesis and LPS biosynthesis proteins. Total sugar intake was strongly correlated with the transporters, ABC transporters, and transcription factors while inversely associated with membrane and intracellular structural molecules and pore ion channels. Sex of participants did not play a significant role in any of the described parameters. Given the importance of SCFAs in gut health, we had a priori interest in “fatty acid biosynthesis” despite its exclusion from the RDA selection process. We found VO_2_peak to be positively correlated (*P* = 0.046, Spearman’s rho = 0.322) with fatty acid biosynthesis (Fig. 6). Thus, to understand which SCFAs correlated with VO_2_peak, we quantified fecal SCFAs via GC.

#### CRF is positively correlated with fecal butyric acid

**Fig. 7:**
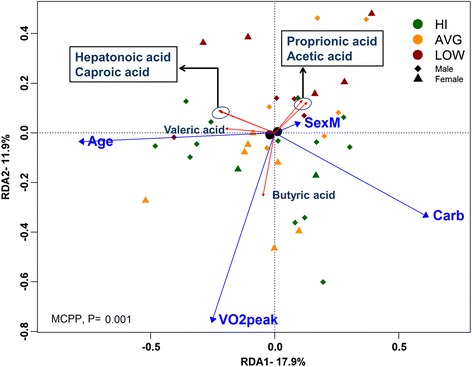
RDA correlation triplot of SCFA abundance data constrained by selected explanatory variables. RDA1 and RDA2 which explain over 29 % of the total variation in SCFA data are plotted. Subjects are color coded according to their CRF for illustrative purposes only as groupings were not included in the model. *Black circles* represent centroids for the categorical variable sex. The global model’s *P* value was calculated using the Monte Carlo Permutation Procedure (MCPP)

RDA triplot corresponding to fecal SCFAs as constrained by our exploratory variables is presented in Fig. 7. The global model selected sex, age, carbohydrate intake, and VO_2_peak as significant (MCPP *P* = 0.001) explanatory variables. Overall, 30.1 % of the total variation of SCFA data could be explained by these variables of which 17.9 and 11.9 % were accounted for by RDA1 and RDA2, respectively. Along RDA1, age was strongly positively correlated with valeric acid and to a lesser degree with hepatonoic and caproic acid, both which were strongly inversely correlated with carbohydrate intake. Along RDA2, VO_2_peak was strongly correlated with butyric acid which is represented mainly across HI and AVG fitness participants. Proprionic and acetic acid on the other hand were inversely correlated to VO_2_peak and were represented across an area with more LOW fitness participants. Sex of the participants as represented by centroids on the triplot did not play a major role in observed variance.

## Discussion

CRF is considered a better predictor of mortality than clinical variables including established risk factors such as smoking, diabetes, and hypertension [32, 33]. Its role as a possible indicator of intestinal microbial diversity, however, has not been investigated. Our regression model showed that ~20 % of variation in gut bacterial alpha diversity could be explained by VO_2_peak alone; in fact, VO_2_peak stood as the only variable that significantly contributed to increased alpha diversity. The primary findings from this study suggest that CRF is a good predictor of gut microbial diversity in healthy humans, outperforming several other variables including sex, age, BMI, and dietary components. Although no single bacterial taxon or group of taxa showed significant variation in abundance in relation to CRF levels, the overall function of the microbiome in high CRF individuals seems to favor an increase in chemotaxis-related genes and decreased LPS biosynthetic pathways. In addition, a strong positive correlation was observed between VO_2_peak and fecal butyric acid, a SCFA associated with gut health [2]. In support of this, when results from the multivariate GLMs were explored without adjustment for multiple testing, abundances of key butyrate-producing members from *Clostridiales*, *Roseburia*, *Lachnospiraceae*, and *Erysipelotrichaceae* genera were found to be significantly associated with VO_2_peak (*P* < 0.05). These results suggest an important role of these taxa in relation to increased butyrate production amongst more aerobically fit individuals; however, future studies should test these ideas under controlled settings.

A recent study by Clarke et al. showed increased gut community richness amongst professional rugby players compared to sedentary BMI-matched and non-matched populations [13]. Due to extreme dietary differences amongst their groups, however, the contribution of physical fitness could not be isolated from possible diet-driven influences. For example, it has been shown that increased species richness as a result of voluntary wheel running in mice is only robust under high-fat but not low-fat feeding conditions [11], highlighting the importance of the background diet. In our study, we minimized the potential influence of diet as a confounding factor by examining LOW, AVG, and HI fitness participants with no significant differences in a comprehensive number of dietary variables. In addition, we quantify fitness using VO_2_peak, a measure of capacity for aerobic work and the gold standard of CRF. In their study, Clarke et al. highlighted the importance of protein intake by showing its positive correlation with alpha diversity. Interestingly, the magnitude of this correlation was comparable to our correlation coefficient between VO_2_peak and alpha diversity in the absence of a correlation between protein intake and alpha diversity. This may suggest that the reported correlation between protein intake and alpha diversity may have been a secondary product of increased CRF amongst the elite athletes. The mechanisms by which physical activity may promote a rich bacterial community are not known but likely involve a combination of intrinsic and extrinsic factors. For example, physically active individuals are more likely to be exposed to their environmental biosphere and follow an overall healthy lifestyle and as so harbor a richer microbiota. Simultaneously, intrinsic adaptations to endurance training can lead to changes in the GI tract, for example, decreased blood flow, tissue hypoxia, and increased transit and absorptive capacity [34, 35]. These and other potential adaptation mechanisms such as change in gut pH may create an environmental setting allowing for richer community diversity.

Beta diversity analysis of our cohort did not show distinct clustering of bacterial communities based on fitness categories. This contrasts with previous reports [11], which showed distinct clustering resulting from wheel running in mice, as well as those by Clarke et al. who showed clustering of rugby players’ microbiota [13]. In addition to extreme dietary differences, several mechanisms may explain these discrepancies. Community clustering amongst cohabited animals or the “cage-effect” is known to show high community structure concordance [36, 37]; it is therefore plausible that this phenomena extends to humans. As team members are likely to spend extended periods of time together on and off the field, there is an increased likelihood of microbial exchange leading to distinct similar bacterial profiles. Participants in the current study on the other hand did not belong to a common organization and did not show any detectable dietary differences. Other components of fitness not accounted for in the current study such as anaerobic capacity and resistance muscle training may also influence community composition, though to date, no existing work has examined these parameters in relation to gut microbiota.

Total protein intake was consistently seen as a significant contributor to beta diversity at each taxonomic rank tested, while sex and age were only influential beyond the phyla level. Unlike dietary carbohydrates and fats, which are commonly studied, the role of protein in the context of intestinal microbiota is considerably less understood. Protein-rich diets have been associated with prevalence of *Bacteroides* genus [38]. Echoing this, results from our RDA analysis showed a strong correlation between protein intake and *Bacteroides* without bias towards any specific fitness groups. Excessive fermentation of dietary protein in the GI tract is generally considered detrimental due to the production of toxic by-products such as amines, phenols, indoles, thiols, and ammonia [39, 40]. Further research however is needed to determine the synthesis kinetics and clinical consequence of these by-products during increased nutritional status and metabolic demands such as during prolonged exercise training. The RDA results further showed significant contribution of members of the *Ruminococcaceae* and *Lachnospiraceae*, two of the most abundant families in gut environments [41], in explaining community diversity. These plant degraders persist in fibrolytic gut communities and are considered an important component of a healthy gut, while their depletion has been observed in IBD patients [42, 43]. *Ruminococcaceae* and *Bacteroides* were anticorrelated, likely reflecting the persistence of these groups in plant carbohydrate- versus protein-rich gut environments, respectively. Interestingly, an unclassified member of the *Christensenellaceae* family was seen significantly correlated with age; this was true despite the limited range of our participants’ age (18–35 years). Though there is limited published work regarding its role, a recent study identified *Christensenellaceae* as the most heritable member of the gut microbiota and highlighted their role in promoting a lean phenotyope [44].

An increase in CRF demands various phenotypic and metabolic adaptations by the host which subsequently may require adaptation by the commensal bacteria. The results of our RDA showed that although VO_2_peak was not significantly associated with variation in community composition, it was associated with changes in the metagenomic functions of the microbiome. Functional categories most strongly correlated with VO_2_peak were related to bacterial motility (categories: bacterial motility proteins, flagella assembly, and bacterial chemotaxis), sporulation, and to a lesser extend the two-component system which enables bacterial communities to sense and respond to environmental factors. One possible mechanism behind these associations may derive from the observation that butyrate, which was more abundant amongst fit participants, can modulate neutrophil chemotaxis [45, 46]. VO_2_peak was inversely correlated with LPS biosynthesis and LPS biosynthesis proteins which were more aligned amongst less fit participants. LPS is a major component of the cell wall of gram-negative bacteria and is considered an endotoxin when present in the blood. By binding to extracellular toll-like receptor 4 (TLR4) found on many cell types, LPS elicits strong inflammatory responses that may be detrimental to the host. Continuous low-level translocation of LPS into circulation can induce chronic low-level inflammatory states that are associated with development of obesity and other metabolic syndromes [47]. These inflammatory states are thought to derive to some extent from inflammatory responses to blood LPS which is elevated in sedentary humans [48]. Exercise training attenuates inflammation in part by reducing elevated blood LPS [48]. The inverse relationship between VO_2_peak and LPS biosynthesis pathways observed in the current study therefore extends previous research, suggesting a beneficial consequence of increased physical activity to derive from decreased LPS biosynthesis. The findings here suggest that the gut microbiota adapt to metabolic demands of a physically active lifestyle, anchored around a set of physiological functions.

Production of SCFAs is the primary result of carbohydrate fermentation under anaerobic conditions in the gut. Butyric acid or butyrate is the most commonly studied of these SCFAs in regard to intestinal health. As the primary food source of colonocytes, butyrate plays an important role in gut homeostasis and health. It has been shown to possess anticancer and anti-inflammatory properties [49] and be involved in gut motility [50, 51], energy expenditure [52], intestinal permeability [53], and appetite control [54], while a decrease in butyrate levels has been suggested in etiology of ulcerative colitis [55]. We observed a strong positive correlation between VO_2_peak and fecal butyrate levels, which could not be accounted for by ingested dietary butyrate or its substrate, fiber. This suggests that the microbial profiles of physically fit individuals favor butyrate producing taxa leading to increased fecal butyrate. This is in accordance with Matsumoto et al. who observed increases in butyrate levels in cecum of rats exposed to 5 weeks of wheel running [10].

## Conclusions

The primary findings from this correlative study suggest that gut microbial diversity in healthy humans is associated with aerobic fitness and that dietary protein moderates microbial community composition. They further suggest that adaptation of the microbiota to demands of increasing physical fitness is anchored around a set of functional cores rather than specific bacterial groups. In particular, the microbiome profile of fit individuals appears to favor butyrate production, a common indicator of gut health, potentially through increases in *Clostridiales*, *Roseburia*, *Lachnospiraceae*, and *Erysipelotrichaceae* genera. Overall, our findings are consistent with a role for physical activity in promoting gut intestinal health via associated changes in the microbial community composition. Based on these findings, we encourage further research on the use of aerobic exercise prescription as an adjuvant therapy in prevention and treatment of dysbiosis-associated diseases.

## Abbreviations

BMI, body mass index; CRF, cardiorespiratory fitness; LPS, lipopolysaccharide; OTU, operational taxa unit; PCA, principal component analysis; RDA, redundancy analysis; SCFAs, short-chain fatty acids; VO_2_peak, peak oxygen uptake

## Additional files

Additional file 1: Sample food survey. A detailed description of all foods and supplemented consumed by the subjects was written during the interview and later analyzed using FoodWorks nutrient analysis software (Version16.0) by a research nutritionist. (PDF 162 kb) Additional file 2: Heyward’s 2006 normal VO_2_max reference chart. Subjects characterized as “Superior” or “Excellent” according to the Heyward classification were grouped under the “HI” group, “Fair” and “Good” subjects were placed into the “AVG” group, and “Poor” was renamed to “LO.” (TIF 2121 kb) Additional file 3: Sampling depth rarefaction curves. Rarefaction curves of all subjects at 97 % similarity levels shown as a function of Shannon diversity index and number of sequence tags sampled. (TIF 823 kb) Additional file 4: Predictor variables included in the RDA models. A manual pre-screening of dietary variables based on existing literature and categories of interest was initially carried. Next, a combination of “both” forward and backward stepwise inclusion selection method using vegan’s *ordistep* function was used on the remaining 23 variables plus VO_2_peak, Sex, BMI, and Age. (TIF 240 kb) Additional file 5: Table summary of PERMANOVA for dietary intake amongst different fitness groups. *df* degrees of freedom, *SS* sum of squares, *MS* mean of squares, *Pr P* value as computed by 999 permutations. (TIF 101 kb) Additional file 6: Correlation matrix of various alpha diversity matrices. A correlation matrix using Spearman’s *r* showing strong correlation between all alpha diversity matrices used. Species richness (S) was thus used as a proxy for the response variable in the multiple regression model. (TIFF 11074 kb) Additional file 7: Beta diversity amongst fitness groups. Three dimensional PCoA plots of genus abundance data transformed with weighted (A) and unweighted (B) unifrac dissimilarity matrices show no clear clustering based on CRF levels. (TIF 256 kb) Additional file 8: Optimal clustering selection of bacterial data. The number of optimal clustering of all data was determined using the Calinski-Harabasz (CH) index. Optimal number of clusters did not identify the classical three enterotypes but rather favored a two cluster partitioning. (TIFF 11074 kb) Additional file 9: Ordination of predicted metagenomic functions data. PCA plot of centered functional category abundance data showing no clear clustering of groups based on their CRF levels. Plots were created using Statistical Analysis of Metagenomic Profiles (STAMP) tool. (TIF 398 kb) Additional file 10: Significant functional categories included in RDA model. A complete list of predicted functional categories and their corresponding RDA 1–4 coordinates determined to be significant in our RDA model. Using a series of Spearman correlations between each category abundance data and RDA1 then RDA2 (alpha adjusted using Bonferroni correction), we identified 65 significant categories out of a total of 274. (DOCX 22 kb)
