# Supplementary material for: Cardiorespiratory fitness as a predictor of intestinal microbial diversity and distinct metagenomic functions
Source: Microbiome. 2016 Aug 8;4:42. doi: 10.1186/s40168-016-0189-7 (PMC4976518; doi:10.1186/s40168-016-0189-7)
Supplement: Additional file 1: — Sample food survey. A detailed description of all foods and supplemented consumed by the subjects was written during the interview and later analyzed using FoodWorks nutrient analysis software (Version16.0) by a research nutritionist. (PDF 162 kb) [file 40168_2016_189_MOESM1_ESM.pdf]

24 Hour Dietary Recall- Filled out Sample

TIME	FOODS<br>BEVERAGES	METHOD OF PREPARATION<br>BRAND NAME	AMOUNT
	EXAMPLE	*WRONG WAY*	
8 AM	Cereal		1 bowl
8 AM	Milk	On cereal	½ glass
8 AM	Juice		1 glass
	Peanut butter and jelly sandwich		1 sandwich
	Apple Juice		1 box
	Candy bar		1
	Hamburger		1
	Soda		1 bottle
	**EXAMPLE**	**RIGHT WAY**	
8 AM	Cereal	Cheerios	1 cup
8 AM	Milk, Whole	On cereal	½ cup
8 AM	Orange Juice	From concentrate	4 oz
12 PM	White Bread	Wonder	2 slices
12 PM	Peanut Butter	Jiff	2 Tbls
12 PM	Grape Jelly	Welch's	1 Tbls
12 PM	Apple Juice	Juice box	8.25 oz
3:30 PM	Candy Bar	Hersheys	1.45 oz
6 PM	Hamburger	McDonald's	1 regular
6 PM	Coke	McDonald's	12 oz

24 Hour Diet Recall

Name: ________________________________ **Date:**________________
Day of the week: Monday Tuesday Wednesday Thursday Friday Saturday Sunday
Does this day represent your typical eating habits? Yes No
Please be as specific and honest as possible for review with the Registered Dietitian. Thank you.

Day 1

Time	FOOD/BEVERAGES	Method of Preparation - (baked, fried, boiled, canned etc.) Brand Name	Amount/Serving Size
