# Supplementary figures and images for: Cardiorespiratory fitness as a predictor of intestinal microbial diversity and distinct metagenomic functions
Source: Microbiome. 2016 Aug 8;4:42. doi: 10.1186/s40168-016-0189-7 (PMC4976518; doi:10.1186/s40168-016-0189-7)

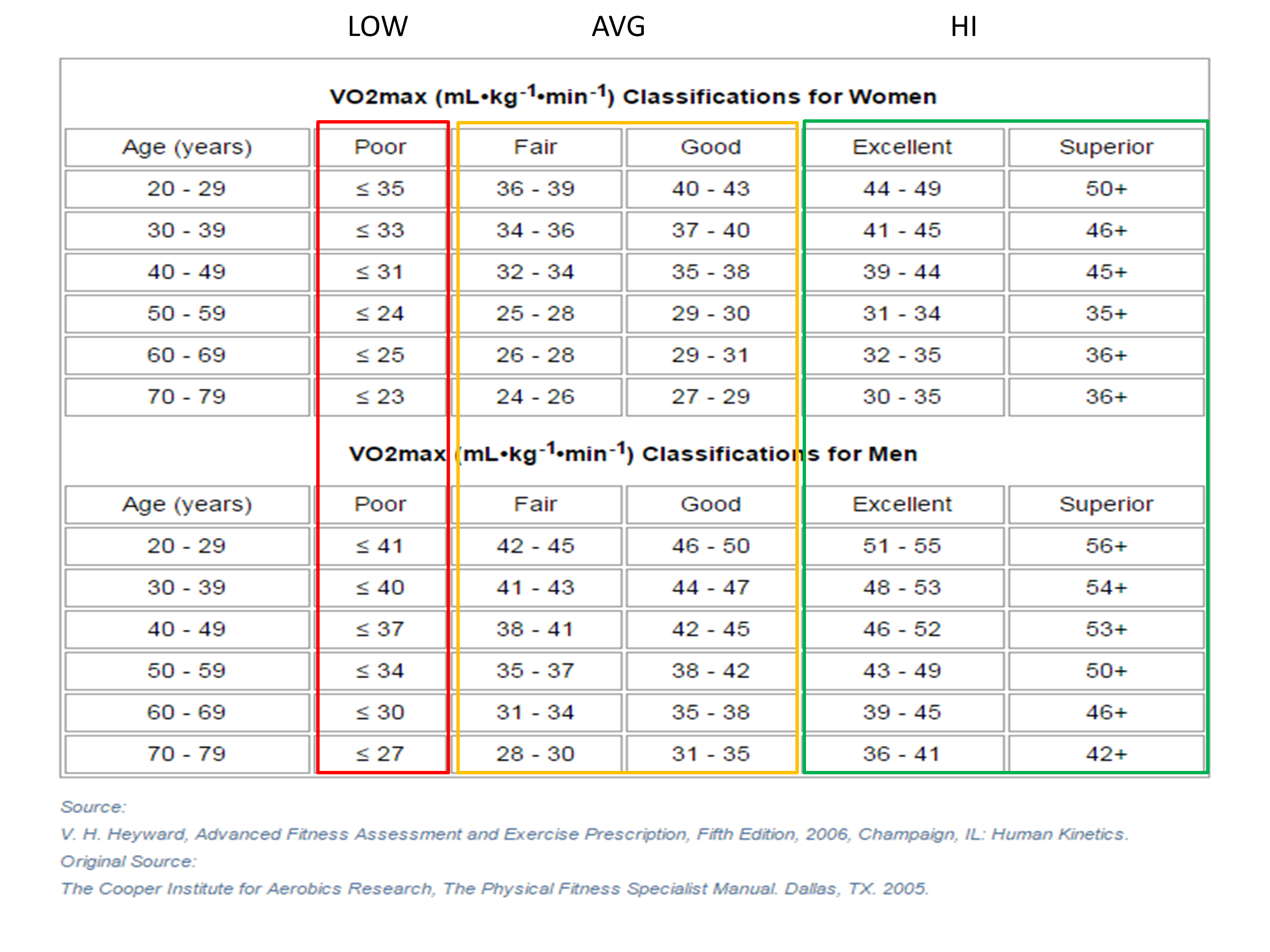

Supplement: Additional file 2: — Heyward’s 2006 normal VO2max reference chart. Subjects characterized as “Superior” or “Excellent” according to the Heyward classification were grouped under the “HI” group, “Fair” and “Good” subjects were placed into the “AVG” group, and “Poor” was renamed to “LO.” (TIF 2121 kb) [file 40168_2016_189_MOESM2_ESM.tif]

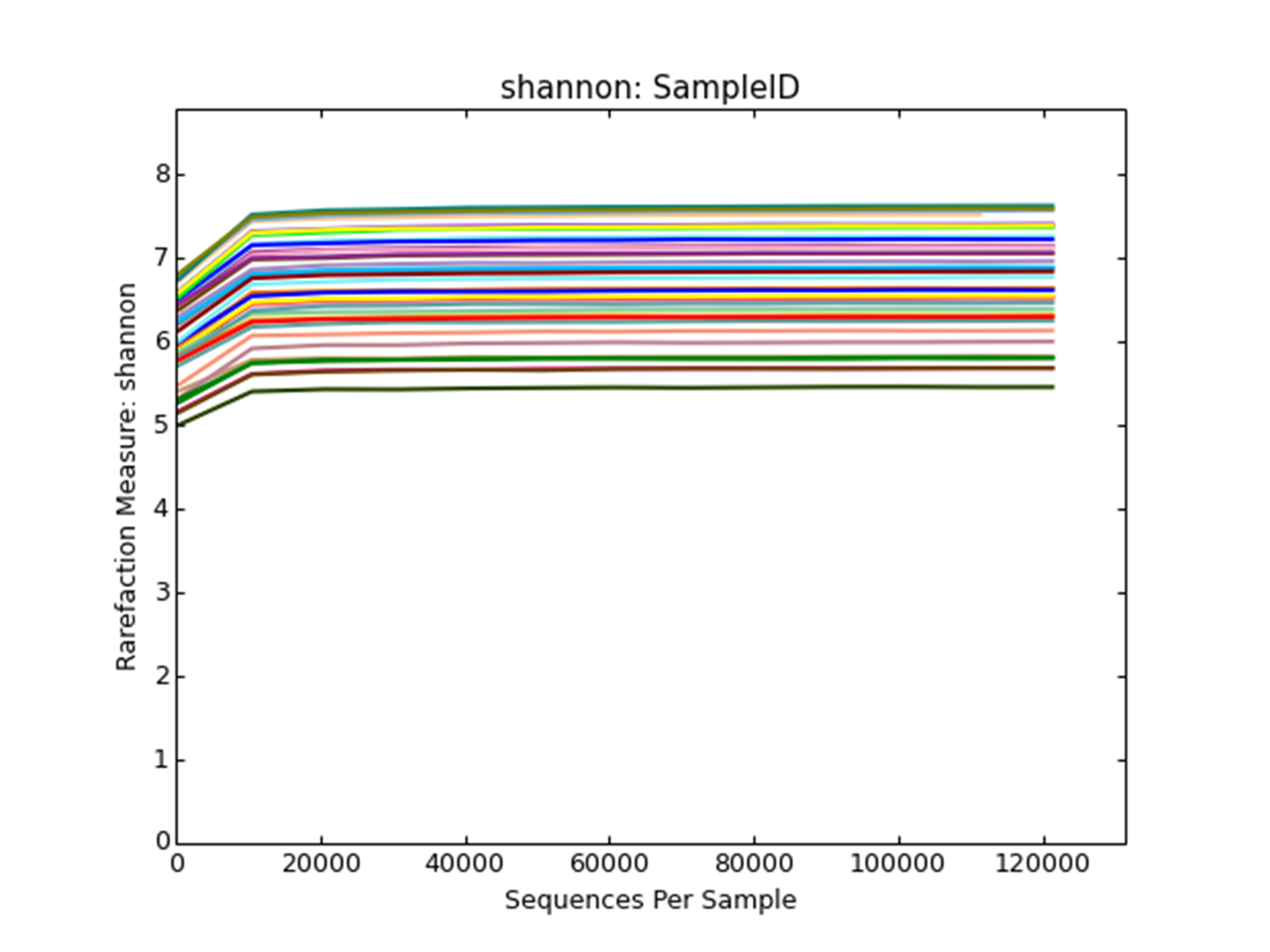

Supplement: Additional file 3: — Sampling depth rarefaction curves. Rarefaction curves of all subjects at 97 % similarity levels shown as a function of Shannon diversity index and number of sequence tags sampled. (TIF 823 kb) [file 40168_2016_189_MOESM3_ESM.tif]

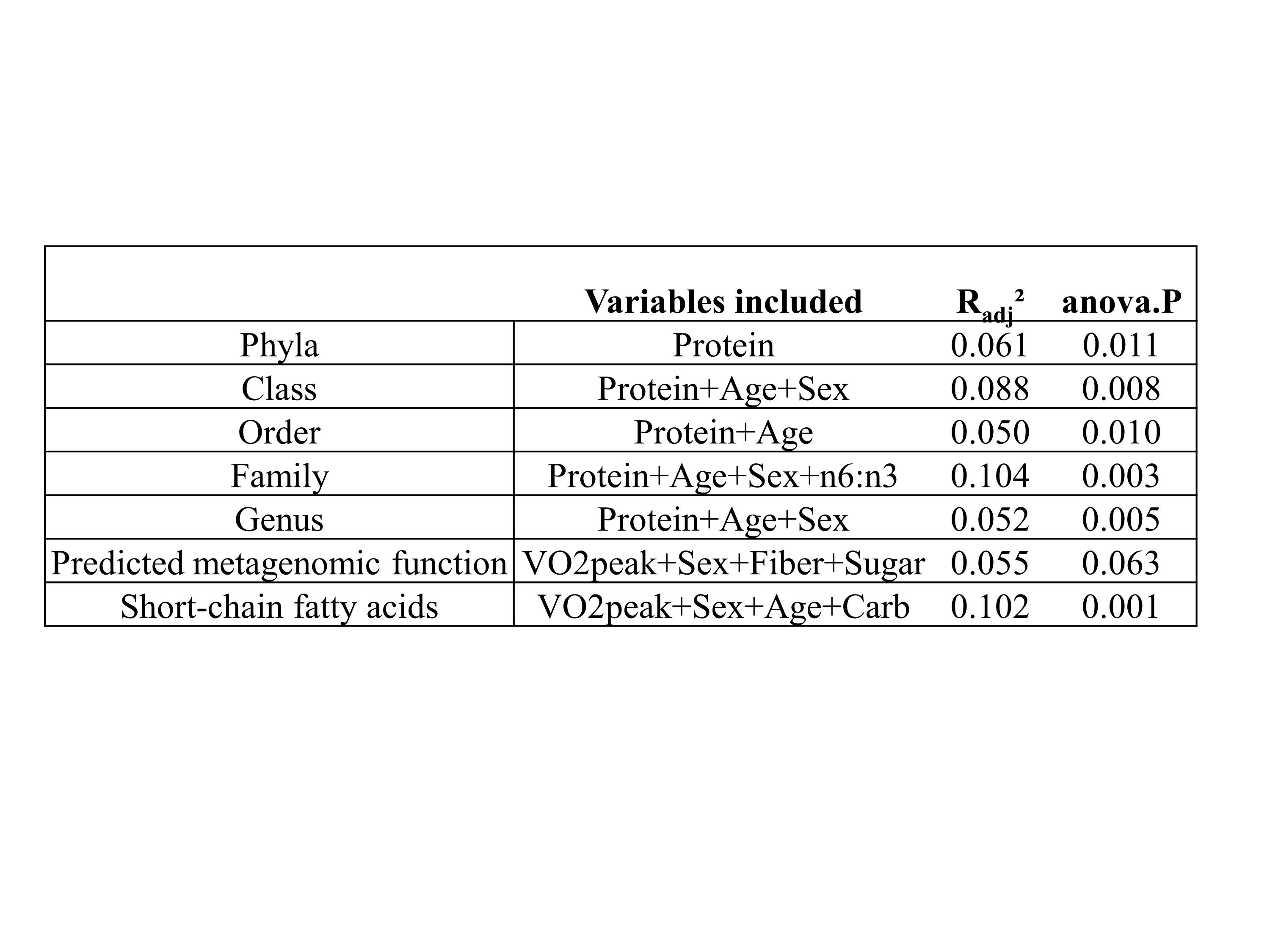

Supplement: Additional file 4: — Predictor variables included in the RDA models. A manual pre-screening of dietary variables based on existing literature and categories of interest was initially carried. Next, a combination of “both” forward and backward stepwise inclusion selection method using vegan’s ordistep function was used on the remaining 23 variables plus VO2peak, Sex, BMI, and Age. (TIF 240 kb) [file 40168_2016_189_MOESM4_ESM.tif]

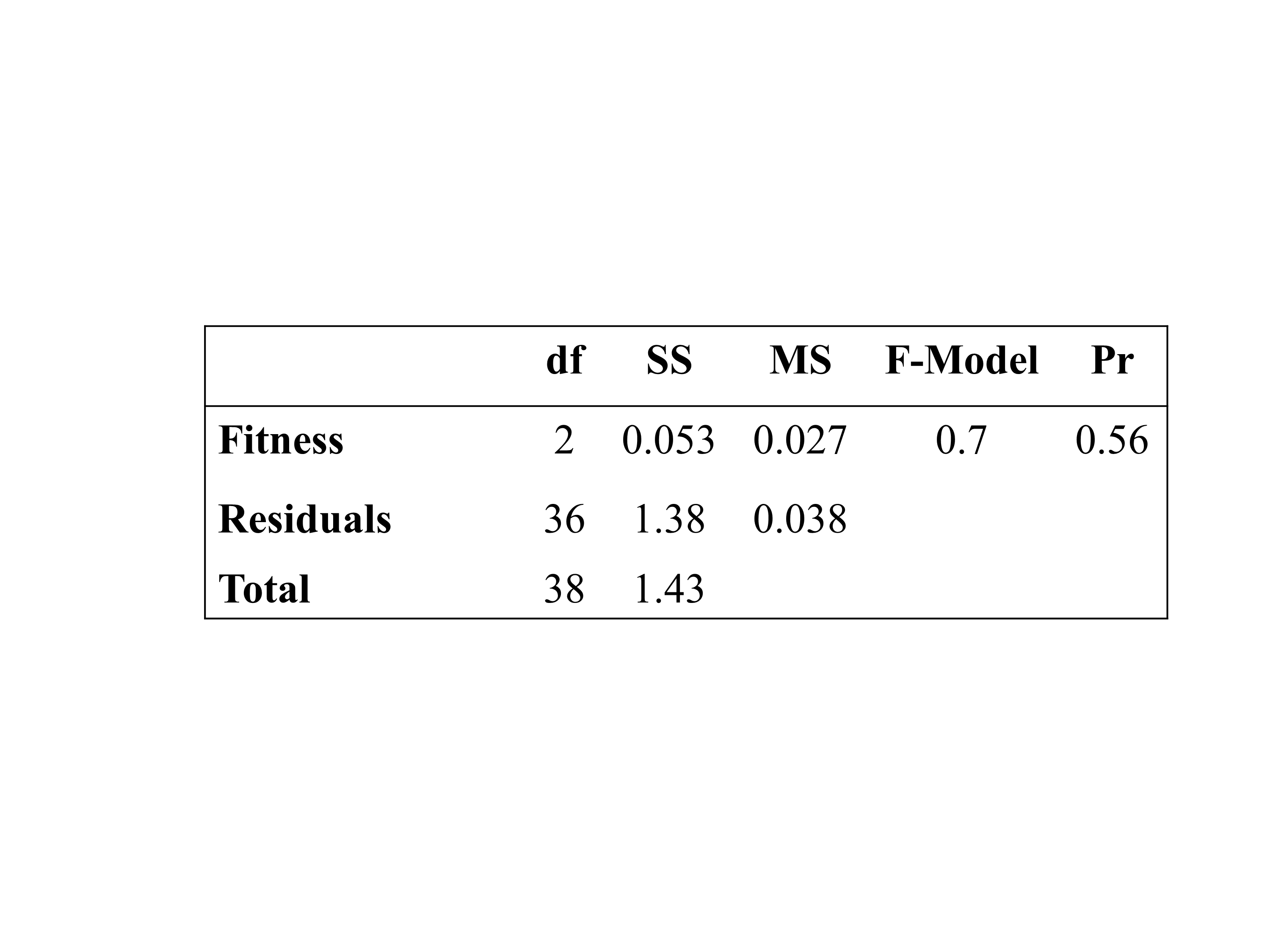

Supplement: Additional file 5: — Table summary of PERMANOVA for dietary intake amongst different fitness groups. df degrees of freedom, SS sum of squares, MS mean of squares, Pr P value as computed by 999 permutations. (TIF 101 kb) [file 40168_2016_189_MOESM5_ESM.tif]

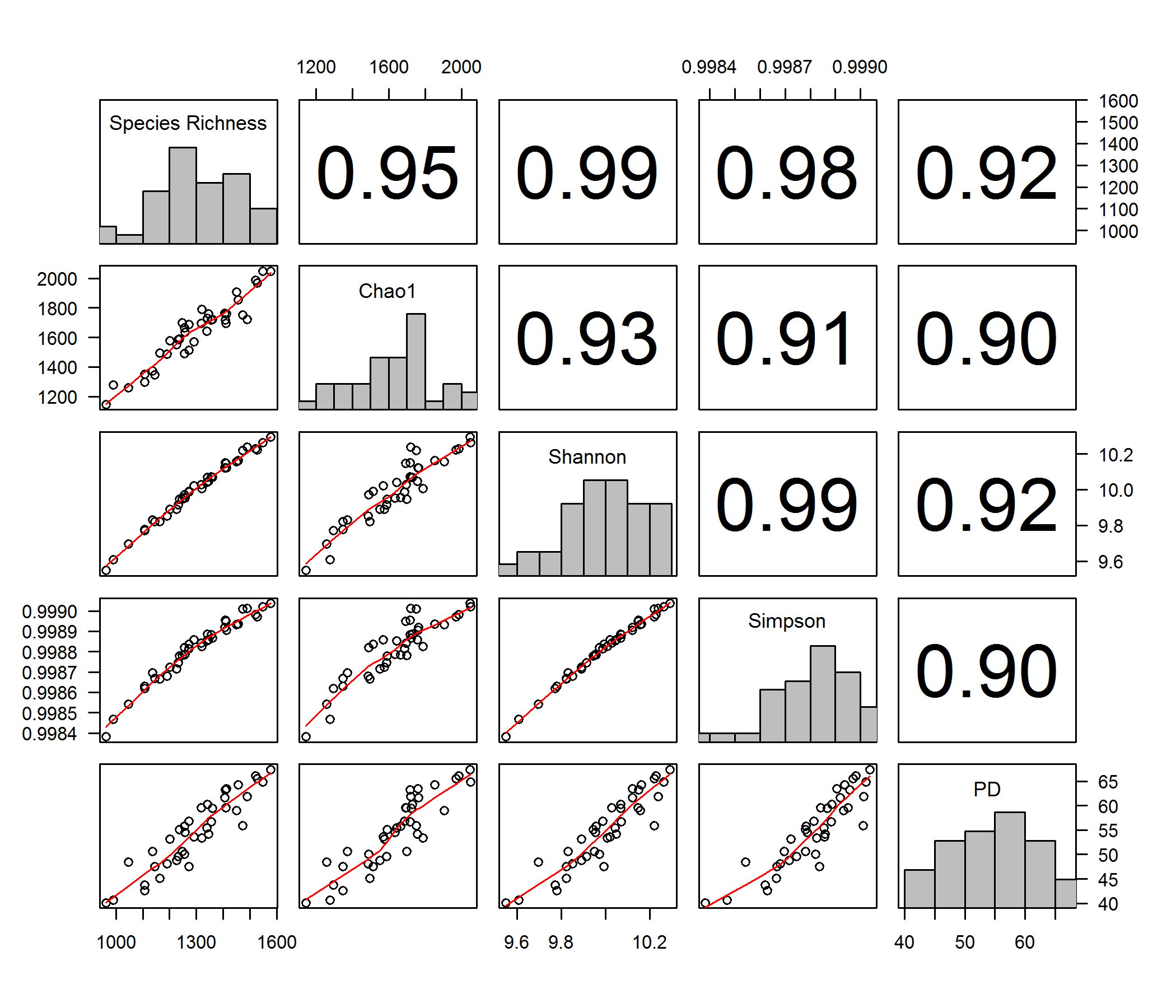

Supplement: Additional file 6: — Correlation matrix of various alpha diversity matrices. A correlation matrix using Spearman’s r showing strong correlation between all alpha diversity matrices used. Species richness (S) was thus used as a proxy for the response variable in the multiple regression model. (TIFF 11074 kb) [file 40168_2016_189_MOESM6_ESM.tiff]

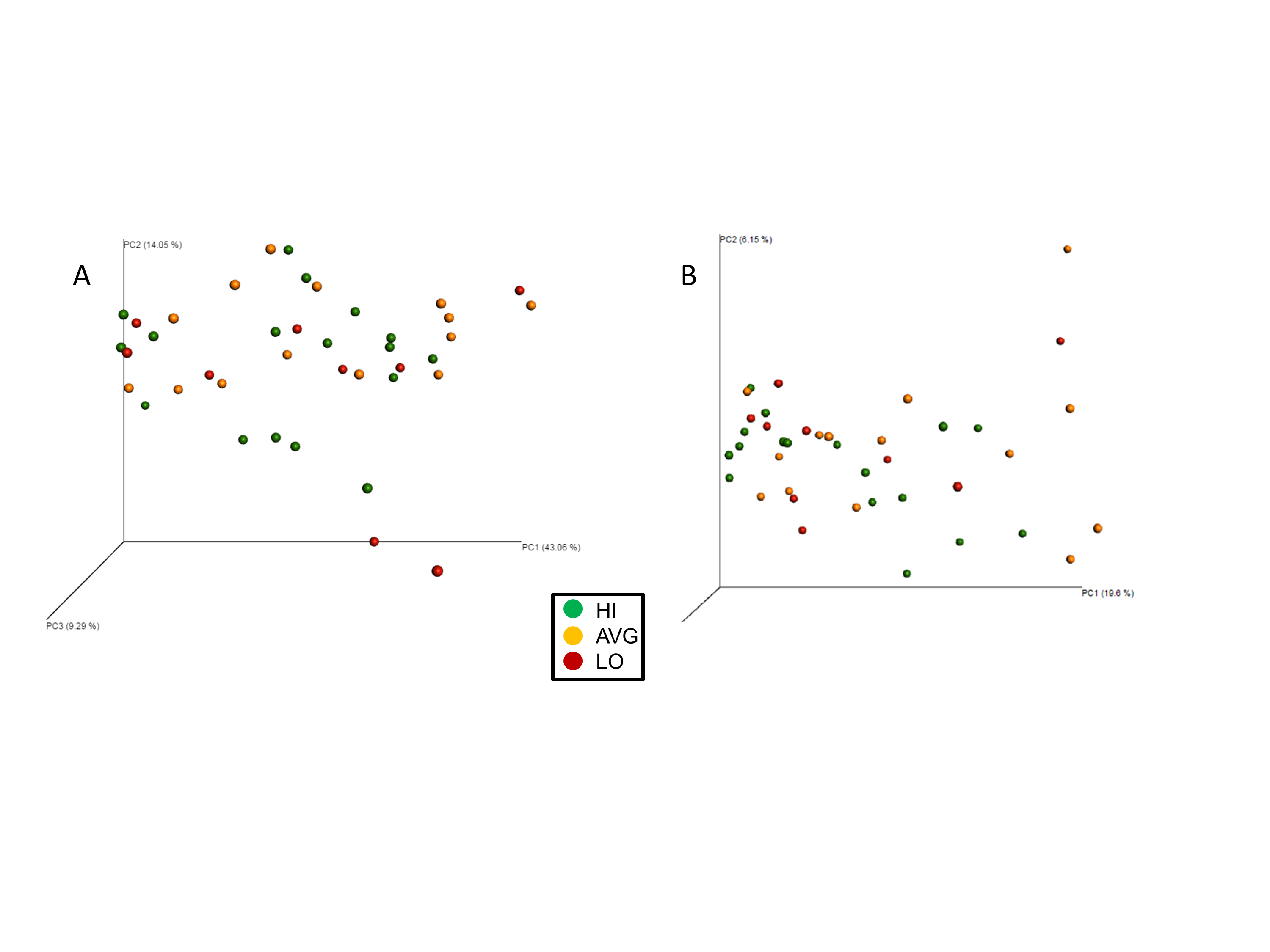

Supplement: Additional file 7: — Beta diversity amongst fitness groups. Three dimensional PCoA plots of genus abundance data transformed with weighted (A) and unweighted (B) unifrac dissimilarity matrices show no clear clustering based on CRF levels. (TIF 256 kb) [file 40168_2016_189_MOESM7_ESM.tif]

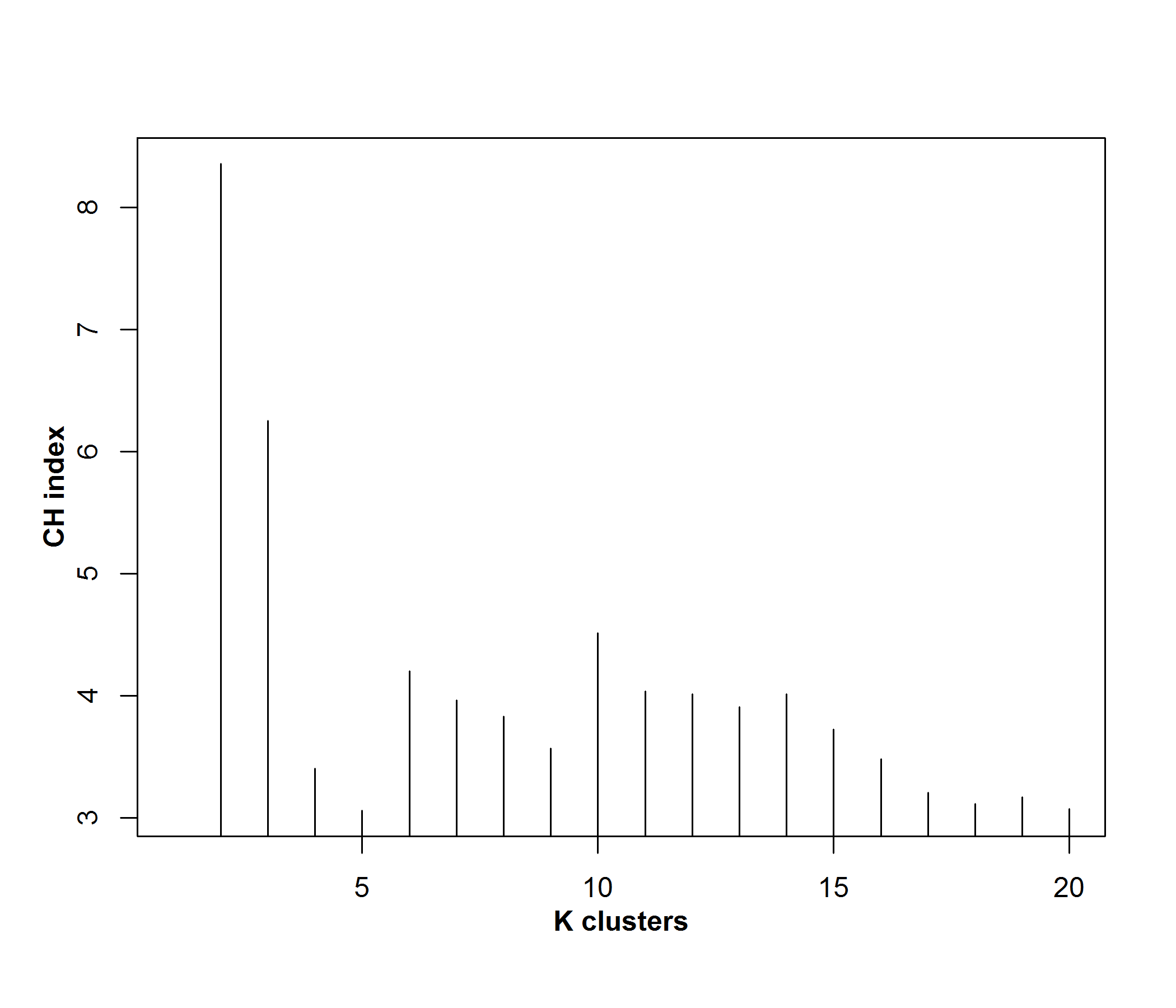

Supplement: Additional file 8: — Optimal clustering selection of bacterial data. The number of optimal clustering of all data was determined using the Calinski-Harabasz (CH) index. Optimal number of clusters did not identify the classical three enterotypes but rather favored a two cluster partitioning. (TIFF 11074 kb) [file 40168_2016_189_MOESM8_ESM.tiff]

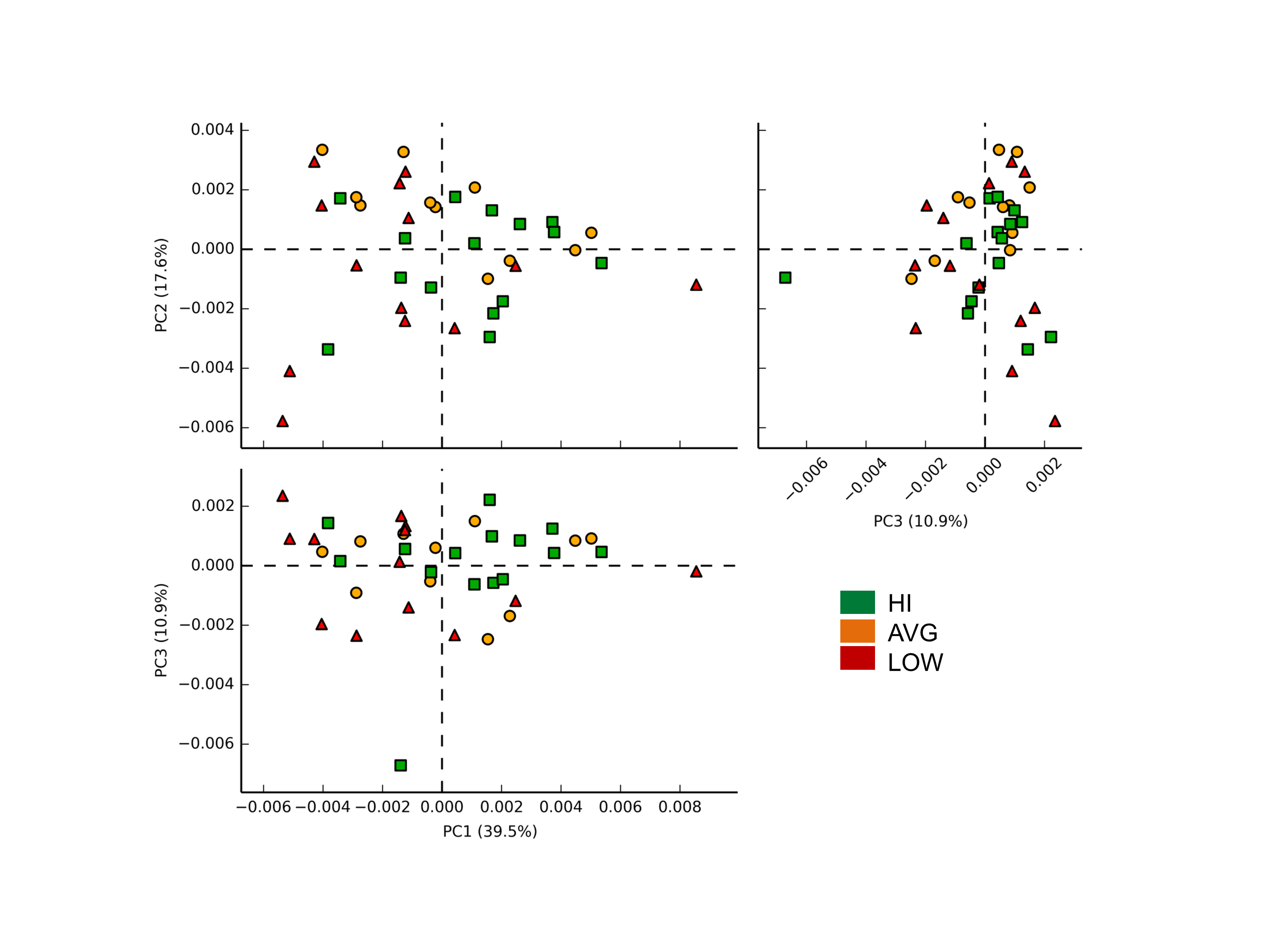

Supplement: Additional file 9: — Ordination of predicted metagenomic functions data. PCA plot of centered functional category abundance data showing no clear clustering of groups based on their CRF levels. Plots were created using Statistical Analysis of Metagenomic Profiles (STAMP) tool. (TIF 398 kb) [file 40168_2016_189_MOESM9_ESM.tif]
