# Supplementary material for: Cardiorespiratory fitness as a predictor of intestinal microbial diversity and distinct metagenomic functions
Source: Microbiome. 2016 Aug 8;4:42. doi: 10.1186/s40168-016-0189-7 (PMC4976518; doi:10.1186/s40168-016-0189-7)
Supplement: Additional file 10: — Significant functional categories included in RDA model. A complete list of predicted functional categories and their corresponding RDA 1–4 coordinates determined to be significant in our RDA model. Using a series of Spearman correlations between each category abundance data and RDA1 then RDA2 (alpha adjusted using Bonferroni correction), we identified 65 significant categories out of a total of 274. (DOCX 22 kb) [file 40168_2016_189_MOESM10_ESM.docx]

| **KEGG Pathway** | **RDA1** | **RDA2** | **RDA3** | **RDA4** |
| --- | --- | --- | --- | --- |
| ABC.transporters | -0.034042554 | 1.65E-02 | 1.43E-02 | -1.84E-03 |
| Adipocytokine.signaling.pathway | 0.012031033 | -3.13E-03 | -1.62E-03 | -1.16E-03 |
| Aminobenzoate.degradation | 0.007751451 | 7.02E-03 | -9.77E-05 | -2.85E-04 |
| Arachidonic.acid.metabolism | 0.013400821 | 1.92E-03 | -4.73E-03 | 1.71E-03 |
| Bacterial.chemotaxis | -0.03658181 | -7.90E-03 | -5.74E-03 | 7.45E-03 |
| Bacterial.motility.proteins | -0.042786454 | -5.23E-03 | -9.92E-03 | 1.34E-02 |
| Biosynthesis.of.ansamycins | -0.006049779 | 2.59E-03 | 2.31E-03 | -1.08E-03 |
| Carbon.fixation.pathways.in.prokaryotes | 0.018106292 | -3.09E-03 | -8.57E-05 | -3.25E-03 |
| Cell.motility.and.secretion | 0.010807989 | 5.26E-03 | -6.35E-04 | -1.45E-03 |
| Cellular.antigens | 0.014521069 | -4.41E-03 | -9.57E-05 | -5.57E-04 |
| Chaperones.and.folding.catalysts | 0.011961607 | -4.79E-03 | -1.97E-03 | 1.76E-04 |
| Citrate.cycle..TCA.cycle. | 0.025186623 | -3.34E-04 | -4.37E-03 | 8.13E-04 |
| Cytoskeleton.proteins | -0.008716167 | -2.09E-03 | 9.62E-04 | -1.45E-03 |
| D.Glutamine.and.D.glutamate.metabolism | 0.003721524 | -2.99E-03 | 1.53E-03 | -2.66E-04 |
| DNA.replication | 0.011770786 | 1.05E-03 | -7.77E-04 | 3.73E-03 |
| Energy.metabolism | 0.010219623 | -2.00E-03 | -4.52E-03 | 1.25E-03 |
| Ethylbenzene.degradation | 0.01144088 | 7.71E-03 | -1.29E-03 | 2.05E-03 |
| Flagellar.assembly | -0.029247024 | -4.49E-03 | -2.90E-03 | 1.21E-02 |
| Folate.biosynthesis | 0.011241505 | -5.03E-03 | -1.34E-03 | 2.25E-04 |
| General.function.prediction.only | 0.003375594 | -2.52E-03 | 2.61E-03 | -1.93E-03 |
| Geraniol.degradation | 0.013149972 | 2.62E-03 | -1.82E-03 | 1.28E-04 |
| Germination | -0.010364449 | -4.01E-03 | -6.40E-03 | -2.97E-04 |
| Glutathione.metabolism | 0.008376741 | -1.75E-03 | 2.81E-03 | -1.38E-04 |
| Glycan.biosynthesis.and.metabolism | 0.019427056 | 6.50E-03 | 1.16E-03 | 8.76E-04 |
| Glycerolipid.metabolism | -0.009904385 | 3.26E-03 | -1.61E-03 | 4.04E-04 |
| Glycine..serine.and.threonine.metabolism | 0.002220289 | -2.64E-03 | 1.47E-03 | 1.19E-04 |
| Glycosaminoglycan.degradation | 0.007663374 | -5.22E-03 | -5.31E-03 | -7.62E-03 |
| Glycosphingolipid.biosynthesis...ganglio.series | 0.013758229 | -6.21E-03 | -2.02E-03 | -5.47E-03 |
| Glycosyltransferases | 0.008375502 | -5.40E-03 | -1.52E-03 | -5.00E-05 |
| Isoquinoline.alkaloid.biosynthesis | 0.005543189 | -2.67E-03 | 3.73E-04 | 2.78E-05 |
| Lipid.metabolism | -0.007009463 | -3.10E-03 | -2.25E-03 | -2.08E-03 |
| Lipopolysaccharide.biosynthesis | 0.047403812 | 4.06E-03 | 7.64E-03 | 6.11E-03 |
| Lipopolysaccharide.biosynthesis.proteins | 0.044080457 | 3.52E-03 | 8.44E-03 | 3.68E-03 |
| Membrane.and.intracellular.structural.molecules | 0.026090326 | -7.47E-03 | -3.88E-03 | -2.28E-03 |
| Metabolism.of.cofactors.and.vitamins | 0.004676421 | -5.56E-04 | -4.32E-03 | 8.56E-05 |
| N.Glycan.biosynthesis | 0.007122247 | -6.20E-04 | -1.45E-03 | 9.26E-04 |
| One.carbon.pool.by.folate | 0.01180214 | -2.91E-03 | 1.49E-03 | 3.09E-03 |
| Oxidative.phosphorylation | 0.011436115 | -3.41E-03 | -2.13E-03 | -2.44E-04 |
| PPAR.signaling.pathway | 0.005627946 | -1.97E-03 | -1.23E-03 | -2.52E-03 |
| Peroxisome | 0.008362242 | -3.45E-03 | 1.51E-04 | -2.12E-04 |
| Plant.pathogen.interaction | -0.00808565 | -3.66E-03 | 1.49E-03 | -4.15E-04 |
| Pores.ion.channels | 0.022120061 | -8.81E-03 | 3.86E-03 | -6.55E-03 |
| Prenyltransferases | 0.013404117 | -1.47E-03 | -2.12E-03 | 1.27E-03 |
| Protein.digestion.and.absorption | 0.013430646 | -5.92E-03 | -7.84E-05 | -9.97E-04 |
| **KEGG Pathway** | **RDA1** | **RDA2** | **RDA3** | **RDA4** |
| Protein.folding.and.associated.processing | 0.009519763 | 1.02E-03 | 6.22E-04 | -3.39E-03 |
| Protein.kinases | -0.013939543 | 1.85E-03 | -1.63E-03 | -7.70E-04 |
| Protein.processing.in.endoplasmic.reticulum | 0.006235997 | -1.79E-03 | -2.59E-03 | -3.27E-03 |
| Purine.metabolism | 0.013105586 | -1.07E-03 | 2.44E-03 | 2.86E-03 |
| RNA.degradation | 0.007634391 | -8.50E-04 | 7.15E-05 | 2.13E-03 |
| RNA.transport | -0.00656263 | -1.82E-04 | 8.35E-04 | -1.01E-03 |
| Riboflavin.metabolism | 0.007051462 | -5.41E-03 | 2.89E-03 | -2.14E-03 |
| Signal.transduction.mechanisms | -0.013356289 | -5.33E-05 | -2.18E-03 | -5.36E-04 |
| Sporulation | -0.043356757 | -3.83E-03 | -7.24E-03 | 5.13E-04 |
| Steroid.hormone.biosynthesis | 0.005215545 | 2.41E-03 | -1.10E-03 | -1.45E-03 |
| Toluene.degradation | 0.023144173 | 6.13E-03 | -3.02E-03 | -2.10E-03 |
| Transcription.factors | -0.022241359 | 1.14E-02 | -3.65E-03 | 3.23E-03 |
| Transporters | -0.059042956 | 2.57E-02 | 1.51E-02 | 2.19E-04 |
| Tropane..piperidine.and.pyridine.alkaloid.biosynthesis | 0.005528516 | 9.21E-05 | 8.36E-04 | 3.15E-04 |
| Two.component.system | -0.025082894 | 2.14E-03 | -5.73E-03 | 7.16E-04 |
| Type.I.diabetes.mellitus | 0.004250365 | -2.87E-03 | -2.05E-04 | -9.67E-05 |
| Ubiquinone.and.other.terpenoid.quinone.biosynthesis | 0.025386109 | 5.15E-04 | -9.53E-03 | 2.36E-03 |
| Valine..leucine.and.isoleucine.degradation | 0.008631266 | 2.32E-03 | 4.77E-03 | -5.64E-04 |
| Vitamin.B6.metabolism | 0.010294613 | 1.04E-03 | -2.55E-04 | 1.52E-03 |
| Zeatin.biosynthesis | 0.006436065 | -1.18E-03 | 4.12E-04 | 1.04E-03 |
